# A Contemporary Systematic Review on Repartition of HPV-Positivity in Oropharyngeal Cancer Worldwide

**DOI:** 10.3390/v13071326

**Published:** 2021-07-09

**Authors:** Amanda F. Carlander, Kathrine K. Jakobsen, Simone K. Bendtsen, Martin Garset-Zamani, Charlotte D. Lynggaard, Jakob Schmidt Jensen, Christian Grønhøj, Christian von Buchwald

**Affiliations:** Department of Otorhinolaryngology, Head & Neck Surgery and Audiology, Rigshospitalet, 2100 Copenhagen, Capitol Region, Denmark; kathrine.kronberg.jakobsen@regionh.dk (K.K.J.); simone.kloch.bendtsen.01@regionh.dk (S.K.B.); martin.garset-zamani@regionh.dk (M.G.-Z.); charlotte.duch.lynggaard@regionh.dk (C.D.L.); jakob.schmidt.jensen.01@regionh.dk (J.S.J.); christian.groenhoej@regionh.dk (C.G.); christian.von.buchwald@regionh.dk (C.v.B.)

**Keywords:** Oropharyngeal cancer, oropharyngeal squamous cell carcinoma, head and neck cancer, human papillomavirus, HPV genotype, prevalence, worldwide, global

## Abstract

Significant variation in human papillomavirus (HPV) prevalence in oropharyngeal squamous cell carcinoma (OPSCC) across countries ranging from 11% in Brazil to 74% in New Zealand has been reported earlier. The aim of this study was to systematically review the most recently published studies on the occurrence of HPV in OPSCC globally. PubMed and Embase were systematically searched for articles assessing the occurrence of HPV+ OPSCC published between January 2016 and May 2021. Studies with a study period including 2015 and the following years were included. Both HPV DNA and/or p16 were accepted as indicators of HPV+ OPSCC. 31 studies were enrolled comprising 49,564 patients with OPSCC (range 12–42,024 patients per study) from 26 different countries covering all continents. The lowest occurrences of HPV+ OPSCC were observed in India (0%) and Spain (10%) and the highest occurrences were observed in Lebanon (85%) and Sweden (70%). We observed great variation in HPV prevalence in OPSCC worldwide varying from 0% to 85%. The highest occurrences of HPV+ OPSCC were found in general in Northern European countries, USA, Lebanon, China, and South Korea. We observed a trend of increase in HPV-positivity, indicating a mounting burden of HPV+ OPSCC.

## 1. Introduction

The global burden of oropharyngeal squamous cell carcinoma (OPSCC) is estimated to be approximately 93,000 new patients per year [1]. For the last decades, the incidence of OPSCC has been increasing due to an increase in human papillomavirus-positive (HPV+) OPSCC [2].

Several HPV genotypes are known as high-risk (HR) genotypes, due to their oncogenic potential. HPV16 is the predominant HR-HPV genotype globally, but other oncogenic genotypes exist (HR non-HPV16) including HPV18, 26, 31, 33, 35, 45, 56, 58, 59 and 67 [3]. The current 9-valent HPV vaccine Gardasil-9^®^ covers low risk (LR) genotypes, that is, HPV6, 11, 16, 18, 31, 33, 45, 52, and 58 but not HPV35 while the 4-valent covers LR HPV6, 11, and HR 16 and 18.

Infection with high-risk (HR) HPV is a well-established risk factor for developing OPSCC and HPV+ OPSCC is associated with lower alcohol consumption, less smoking, fewer comorbidities, and younger age versus HPV− OPSCC [4,5].

HPV+ OPSCC has a better prognosis compared to HPV− OPSCC and demonstrates distinct clinical, histopathological, and genetic characteristics [6,7,8].

p16 is often overexpressed in HPV+ OPSCCs and can therefore be used as a surrogate marker for infection with active HPV, but double positivity for p16 by immunohistochemistry (IHC) and HPV DNA has shown better prognostication [8,9]. However, there is a remarkable variation in detection methods of HPV+ OPSCC across studies [10,11].

Significant variation in HPV prevalence in OPSCC across countries with HPV positivity ranging from 11% in Brazil to 74% in New Zealand has been reported earlier [10]. It is important to better characterize the current influence of HPV+ OPSCC globally to provide useful information for clinicians and to expand and design the HPV vaccination programs to prevent HPV+ OPSCC at a global level. The aim of this study was to systematically review the most recently published studies on the HPV prevalence in OPSCC worldwide.

## 2. Materials and Methods

### 2.1. Search Strategy and Data Extraction

In May 2021, one author (ALC) systematically searched PubMed and Embase for articles assessing the prevalence of HPV+ OPSCC published between January 2016 and May 2021. Studies with a study period including 2015 and the following years were enrolled. Only studies with data regarding HPV status and with a minimum of five cases were included. Studies evaluating the HPV prevalence in specific subpopulations stratified by gender, comorbidities, or ethnicity were excluded. Both HPV DNA and/or p16 were considered as indicators of HPV+ OPSCC.

The following search strategy was used when searching PubMed: (Oropharyn* cancer or oropharyn* neoplasm or oropharyn* carcinoma or oropharyn* malignancy or oropharyn* tumour or oropharyn* tumor) AND (HPV or human papillomavirus or human papilloma virus or papillomaviridae) AND (incidence or frequency or prevalence). MeSH terms were included as well: Papillomaviridae, neoplasm, carcinoma, incidence, and prevalence. The search was limited to the English language and articles published between January 2016 and May 2021.

The same keywords were used to create three different searches in Embase, which were combined with “AND”. The searches were restricted to English language, human studies, and articles published between 2016–2021:

1. HPV or human papillomavirus or human papilloma virus
2. Oropharyn_ cancer or oropharyn_ neoplasm or oropharyn_ carcinoma or oropharyn_ malignancy or oropharyn_ tumor or oropharyn_ tumour
3. Incidence or frequency or prevalence

The subsequent parameters were evaluated and extracted from the studies: anatomical sublocation, HPV status, definition of HP-positivity, HPV genotypes, smoking, TNM-stage, age, and sex.

### 2.2. Data Analysis

The occurrences of HPV+ OPSCC were expressed as relative frequencies. A proportion meta-analysis was made to evaluate the overall pooled HPV prevalence and heterogeneity between studies in relation to study size. A random-effect model was considered due to high heterogeneity, with an I2 test value >75% and many small sample sizes. Statistics were performed in RStudio using the packages “metafor”, “meta”, and “forestplot”.

## 3. Results

**Figure 1:**
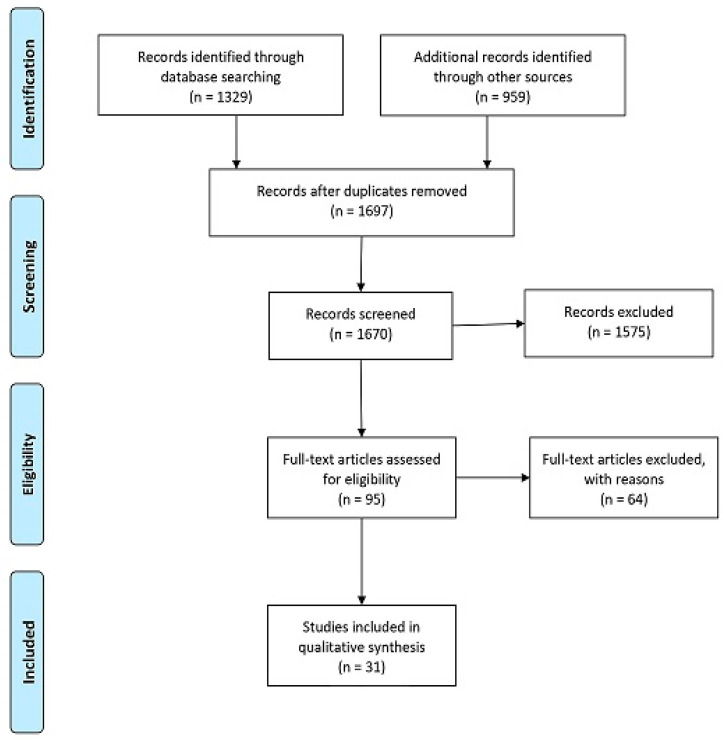
PRISMA flow diagram of article selection.

The PubMed and Embase search generated a total of 1697 studies, Figure 1. Thirty-one studies met the inclusion criteria comprising 49,564 patients with OPSCC representing 26 different countries covering South America, the USA, Africa, The Middle East, Asian-Pacific, and Europe.

### 3.1. Global Burden of HPV+ OPSCC

The study population varied from 12 in a study from Ghana [12] to 42,024 in the USA [13], Table 1. Most of the minor studies were observed in Middle Eastern and African countries (*n* = 12–34). HPV-positivity varied from 0% in India [14] to 85% in Lebanon [15]. Both high and low HPV prevalence were observed across all geographical areas, Table 1.

**Figure 2:**
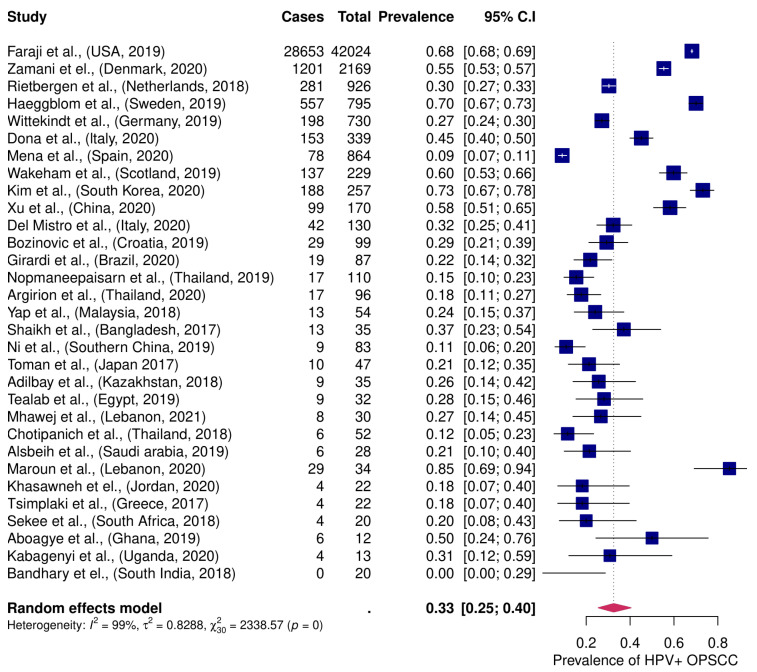
Proportion meta-analysis of HPV+ OPSCC in enrolled studies. Occurrence of HPV+ OPSCC worldwide among patients with OPSCC. OPSCC: oropharyngeal squamous cell carcinoma. HPV +: human papillomavirus-positive.

In the proportional meta-analysis, the pooled prevalence of HPV+ OPSCC was 33 % (95 %CI; 25–40 %). Great heterogeneity regarding study size was observed between enrolled studies, *I*^2^ > 75%, Figure 2.

HPV-positivity according to oropharyngeal subsites was reported in 19 studies (48,250). Ten studies reported the highest HPV+ rate in palatine tonsillar squamous cell carcinoma (TSCC) (*n* = 47,690) [4,19,20,21,28,32,33,34,35,36], and three reported the highest HPV+ rate in base of tongue squamous cell carcinoma (BTSCC) (*n* = 2 51) [29,37,38].The proportion of HPV-positivity among patients with TSCC varied from 15% to 86%, among patients with BTSCC from 1% to 82%, and for other oropharyngeal locations from 1% to 100%, Table 1.

**Figure 3:**
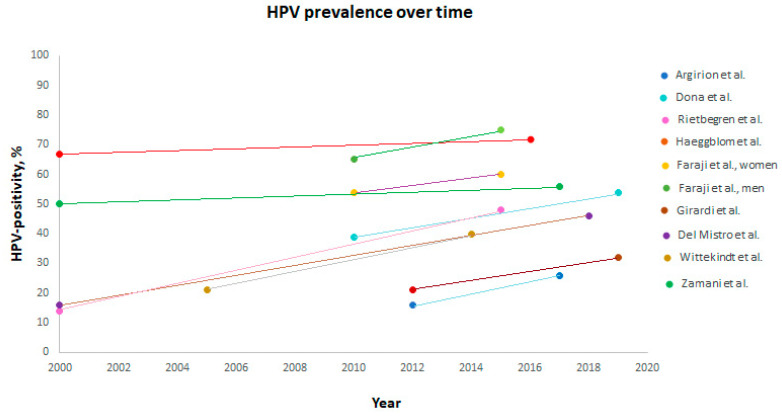
HPV-positivity in patients with OPSCC over time worldwide. OPSCC: oropharyngeal squamous cell carcinoma. HPV: human papillomavirus.

Nine studies demonstrated an increase in HPV prevalence over time (*n* = 47,296) [4,13,19,31,32,33,35,38,39]. An increase was seen in Thailand (16% in 2012 to 26% in 2017) [19], USA (54% in women and 65% in men in 2010 to 60% and 75% in 2015, respectively) [13], Germany (doubled from 2005 to 40% in 2014) [39], Netherlands (14% in 2000 to 48% in 2015) [35], Denmark (50% in 2000–2010 to 56% from 2015–2017) [4], Italy (40% in 2010–2014 to 54% in 2015–2019 as reported by Dona et al. and from 16% in 2000–2006 to 46% from 2013–2018 as reported by Del Mistro et al.) [32,38], Brazil (21% in 2012 to 32% in 2019) [31], and Sweden (67% in 2000–2004 to 72% in 2013–2016) [33], Figure 3.

### 3.2. HPV Detection Methods and Definition

HPV detection methods varied among the included studies. Fifteen studies were based on double positivity with both HPV DNA PCR and p16 IHC (*n* = 6624) [4,16,19,20,22,23,28,30,32,33,34,35,38,39,40], eight studies were based on HPV DNA alone (*n* = 426) [12,14,15,24,25,29,36,41], six on p16 alone (*n* = 391) [17,18,21,26,27,31], one study was based on both HPV DNA and HPV RNA (*n* = 99) [37] and one study did not report a detection method (*n* = 42,024) [13]. Most studies using p16 IHC defined p16-positivity (p16+) according to ASCO guidelines with ≥70% positive staining [42] (*n* = 2663), but one study defined p16+ as ≥10% positive staining (*n* = 30) [30], four studies defined p16+ as ≥75% positive staining (*n* = 2678) [4,22,31,32] and one study did not account for p16-positivity (*n* = 926) [35]. Both high HPV-prevalence and low HPV-prevalence were found in studies regardless of detection method. Table 1.

### 3.3. HPV Genotypes

Fifteen studies (*n* = 4294) evaluated infection with specific HPV genotypes [12,15,18,19,20,21,22,25,28,29,32,34,38,40]. In all studies, HPV16 was the most predominant subtype and was observed in up to 100% of HPV+ OPSCC cases [12,18,28,29]. Other common genotypes observed were HPV18 (1–12%) [4,15,19,20,21,22,32,34,38,40], HPV33 (1–7%) [4,20,21,32,34,38] and HPV35 (3–5%) [4,20,32,34]. Co-infections were reported in 23 cases in total of which HPV16 was present in most cases and was co-infected with HPV18 (*n* = 4), HPV52 (*n* = 2) and HPV59 (*n* = 1) [15,19,20,22]. One study did not report the distribution of genotypes in co-infected cases (*n* = 16) [4].

### 3.4. Clinical Characteristics of HPV+ OPSCC

Nine studies (*n*= 45,603) observed a lower mean or median age in HPV+ OPSCC ranging from 56–62 years [4,13,19,21,26,27,30,31,35], while one study observed a higher mean age in HPV+ OPSCC [15]. The mean/median age among HPV− OPSCC ranged from 57–67 years. Eighteen studies did not report data on age. Table 1**.**

The female to male ratio varied from 1:1 in Egypt to 1:12 in Thailand among patients with OPSCC, see Table 1 [16,24]. In eight studies (*n* = 3867), HPV+ OPSCC were associated with less smoking, whereas smoking was not associated with HPV-status in two studies [4,15,21,24,26,30,31,32,35,37]. Four studies did not report any differences regarding TNM-stage when stratifying for HPV-status (*n* = 207) [15,19,26,30], while seven studies reported a higher N-stage for HPV+ OPSCC compared to HPV− OPSCC (*n* = 46,522) [4,13,21,32,33,35,37] and seven studies demonstrated a lower T-stage among HPV+ OPSCC than HPV− OPSCC (*n* = 46,352) [4,13,31,32,33,35,40]. Nineteen studies did not account for TNM-stage in relation to HPV-status (2748).

## 4. Discussion

This systematic review investigated the global occurrence of HPV+ OPSCC. We enrolled 31 studies comprising 49,564 patients with OPSCC from 26 different countries covering all continents. The HPV prevalence varied from 0% to 85% [14,15]. The lowest proportions of HPV+ OPSCC were seen in India and Spain, while the highest proportions of HPV+ OPSCC were observed in South Korea and Lebanon [15,20]. HPV+ OPSCC was more prevalent than HPV− OPSCC in Sweden, Denmark, Scotland, China, South Korea, Lebanon, and the USA [4,13,15,20,21,33,41]. A higher occurrence of HPV+ OPSCC in northern European countries than in outhern European countries has also been reported previously [11,43]. Twenty-two of the included studies observed a greater incidence of HPV− OPSCC than HPV+ OPSCC, distributed in Europe, South America, Africa, The Middle East, and Asia-Pacific [14,16,17,18,19,22,23,24,25,26,27,28,29,30,31,32,34,35,36,37,38,39,40]. The lowest HPV prevalence was observed in India (0%), but this study comprised only 20 patients and had a very high frequency of alcohol (80%), smoking (80%), betel nut chewing (75%), and beedi smoking (90%) [14]. The highest HPV prevalence was observed in Lebanon (86%), which comprised only 34 patients and was based on HPV DNA alone [15]. Another Lebanese study found a HPV prevalence of 27% which included 30 patients and HPV status was based on HPV DNA and p16+ (>10%) [30].

Seventeen of the enrolled studies provided sufficient information on anatomical sublocations, and the rate of HPV-positivity varied greatly according to anatomical subsite [4,13,15,19,20,21,22,24,28,29,32,33,34,35,36,37,38]. Overall, most studies observed the highest HPV prevalence in TSCC and BTSCC and the lowest prevalence in other oropharyngeal subsites, but two of the studies with the highest proportion (Haegblomm et al. and Zamani et al.) only included TSCC and BTSCC [4,33].

When studies addressed other oropharyngeal sites besides TSCC and BTSCC, the overall HPV prevalence would therefore be lower than if only TSCC/BTSCC were included. Additionally, 12 studies did not address anatomical sublocations [12,14,16,17,18,25,26,27,31,39,40,41]. This variation in reporting and including specific anatomical subsites complicates comparison between studies. This is in line with Stjernstrøm et al., who also observed differences in how European studies reported and included oropharyngeal sublocations [11].

Nine studies revealed an increase in HPV prevalence over time (*n* = 47,296) [4,13,19,31,32,33,35,38,39]. Of note, an increase was also observed in countries who have previously reported a lower HPV prevalence, e.g., Germany (nearly doubled from 2005 to 40% in 2014) [39], Netherlands (14% in 2000 to 48% in 2015) [35], Thailand (16% in 2012 to 26% in 2017) [19], Italy (40% in 2010–2014 to 54% in 2015–2019 as reported by Dona et al. and from 16% in 2000–2006 to 46% from 2013–2018 as reported by Del Mistro et al.) [32,38], and Brazil (21% in 2012 to 32% in 2019) [31]. The pooled burden of HPV+ OPSCC was 33% (95% CI 26%-41%) globally, which is lower than previously reported [10]. However, this review did not include studies from Middle Eastern or African countries. This emphasizes that HPV+ OPSCC is an increasing health burden not only in western countries but also in Asia-Pacific and South America.

More than 100 countries have introduced the HPV vaccine in vaccination programs, but primarily to prevent cervical cancer, and is, therefore, most offered to young girls alone [44]. To reach herd immunity, more than 80% HPV vaccine coverage amongst girls is needed, but gender-neutral vaccination programs have proven to be more robust in reaching stable high HPV vaccine coverage protecting both females and males [45,46,47]. Several countries have included young boys in the HPV vaccine programs, e.g., North America, Denmark, Netherlands, and the UK. The 9-valent vaccine covers most of the HR-HPV genotypes causing HPV+ OPSCC globally observed in this review, including HPV16, HPV18, and HPV33 but not HPV35.

The lack of consensus on how to detect HPV was striking, and only 15 studies used double positivity based on both p16 and HPV DNA [4,16,19,20,22,23,28,30,32,33,34,35,38,39,40], and one study was based on both HPV DNA and HPV RNA [37] (*n* = 6723) to define HPV+ OPSCC. It has been shown that evaluating only p16 overexpression or HPV DNA has a lower specificity compared to the combination of the two [48]. Incongruence between p16+ and HPV+ has been revealed and can result in false-positive or false-negative results. HPV+/p16- OPSCC might be due to a HPV bystander infection and not a HPV+ cancer, while on the other hand, HPV-/p16+ OPSCC may be caused by genetic alterations and not active HPV infection. But both high and low HPV prevalence were seen regardless of the detection method used.

Most of the studies using p16 ICH defined p16-posititivity according to ASCO guidelines with >70% staining (*n* = 2663). Four studies defined p16+ as ≥75% staining (*n* = 2678) [4,22,31,32], while one study defined p16+ as less, ≥10% staining (*n* = 30) [30]. The largest study enrolled, comprising 42,924 patients from the USA, did not report how they defined HPV+ OPSCC [13]. Mariz et al. has also recently showed discrepancies in the definition of p16-positivity in a systematic review [10]. In order to assess the global burden of HPV+ OPSCC and to compare studies, it is important to align HPV detection methods to identify true, active HPV-infection in OPSCCs.

This review was based on data from a very heterogeneous population of studies derived from different countries, with varying health systems and with different burdens of smoking and alcohol consumption. There was great variety in how the studies reported anatomical subsites within the oropharynx and HPV detection methods. Additionally, there was great variation in the study sizes (12–42,024) and with a tendency towards small study populations from countries with a previously less described impact of HPV in OPSCC, e.g., African and Middle Eastern countries (*n* = 12–34), whereas studies from Europe and the USA were greater in size (*n* = 22–42,024). These differences may influence the distribution of HPV+ OPSCC observed. Bigger studies from especially African and Middle Eastern countries are needed to further address the burden of HPV+ OPSCC in these geographical areas.

To our knowledge, this is the biggest systematic review on the global burden of HPV+ OPSCC including geographical areas, that have not previously been described in a systematic review. We observed a pooled burden of HPV+ OPSCC of 33% (95% CI 25–40%) globally and, to our knowledge, the so far described highest occurrence of HPV+ OPSCC of 86% in Lebanon.

## 5. Conclusions

In conclusion, in this systematic review, we observed great variation in the prevalence of HPV+ OPSCC worldwide, i.e., varying from 0% in India to 86% in Lebanon among 31 enrolled studies comprising 49,564 patients from 26 countries. In numerous studies, an increase in HPV prevalence was observed over time, indicating an increasing burden of HPV+ OPSCC worldwide. HPV16 was the predominant genotype, but also HPV18, 33, and 35 were frequent. HPV16, 18, and 33 are covered by the 9v HPV vaccine. Inconsistencies in HPV detection methods observed emphasize the need for a more uniform definition of HPV positivity.

## Figures and Tables

**Table 1:** HPV-positivity in OPSCC among patients worldwide.

Authors (Area, Publication Year)	Centre/Data Source	Study Period	Cases	Age Median			F:M Ratio in Total	Share of HPV+ Patients in %	OPSCC Sublocations (Share of HPV+ Patients in %)	Definition of HPV Positivity Based on
				HPV+	HPV-	Overall				
**Africa**										
Ghana, 2019 [12]	Tertiary Hospital, Kusami	2007–2016	12	-	-	-	-	50	-	HPV DNA
Egypt, 2019 [16]	National Cancer institute	2008–2015	32	-	-	-	1:1	28	-	HPV DNA and p16 (>70%)
Uganda, 2020 [17]	Uganda Cancer Institute	2018–2019	13	-	-	-	-	31	-	p16 (>70%)
South Africa, 2018 [18]	Universitas Academic Hospital	2014–2017	20	-	-	-	-	20	-	p16 (>70%)
**Asian-Pacific**										
South India, 2018 [14]	K.S.Hegde Medical Academy	2014–2016	20	-	-	-	-	0	-	HPV DNA
Thailand, 2020 [19]	Srinagarind Hospital	2012–2017	96	55 *	57 *	-	1:4.6	18	TSCC (41%), BTSCC (7%) soft palate, other (4%)	HPV DNA and p16 (>70%)
South Korea, 2020 [20]	Catholic Medical Center hospitals, Seoul St. Mary’s Hospital, Bucheon St. Mary’s Hospital	2011–2019	257	-	-	-	-	73	TSCC (78%), BTSCC (61%), soft palate, uvula, other (38%)	HPV DNA and p16 (>70%)
China, 2020 [21]	Fudan University Shanghai Cancer Center	2007–2019	170	56	59	-	1:6	58	TSCC (69%), BTSCC (42%), soft palate, pharyngeal wall (27%)	p16 (>70%)
Southern China, 2019 [22]	Foshan First People’s Hospital, First Affiliated Hospital of Guangdong Pharmaceutical University, Second Affiliated Hospital of Wenzhou Medical University	2009–2017	83	-	-	-	1:4	11	TSCC (48%), BTSCC (19%), palate (50%)	HPV DNA and p16 (>75%)
Thailand, 2019 [23]	King Chulalongkorn Memorial Hospital	2010–2016	110	59 *	59 *	59 *	1:6	15	TSCC (35%)	HPV DNA and p16 (>70%)
Thailand, 2018 [24]	Chonburi and Lopburi cancer hospitals	2016	52	-	-	60.4	1:12	12	TSCC (9%), BTSCC (1%), other (16%)	HPV DNA (only 16/18)
Bangladesh, 2017 [25]	Dhaka Medical College Hospital	2014–2016	35	-	-	-	-	36	-	HPV DNA
Japan, 2017 [26]	Nihon University, Kurume University	2010–2015	47	57 *	64 *	-	1:8	22	-	p16 (>70%)
Malaysia, 2018 [27]	-	2004–2015	54	62 *	67 *	65 *	1:2	24	-	p16 (>70%)
**Middle East**										
Lebanon, 2020 [15]	American university og Beirut Medical Center	1972–2017	34	59 *	58 *	59 *	1:3	85	TSCC (86%), BTSCC (82%), soft palate (100%)	HPV DNA
Saudi Arabia, 2019 [28]	KFSHRC Hospital	2002–2016	28	-	-	-	1:1.8	21	TSCC (25%, BTSCC (21%), soft palate (1%)	HPV DNA and p16 (>70%)
Jordan, 2020 [29]	King Hussein Cancer and Medical Centers	2013–2018	22	-	-	-	-	18	TSCC (33%), BTSCC (50%), soft palate (33%)	HPV DNA
Lebanon, 2021 [30]	Hotel Dieu de France Hospital	2010–2016	30	60 *	64 *	58 *	1:2	27	lymfoid areas (77%), non-lymfoid areas (23%)	HPV DNA and p16 (>10%)
**North America**										
USA, 2019 [13]	United States National Cancer Database	2010–2015	42.024	59 *	62 *	60 *	1:4.8	68	TSCC(72%), BTSCC (67%), other (50%)	-
**South America**										
Brazil, 2020 [31]	Ana Nery Hospital	2017–2019	87	61 *	62 *	61 *	1:6	21	-	p16 (>75%)
**Europe**										
Denmark, 2020 [4]	Rigshospitalet, University of Copenhagen	2000–2017	2169	61 **	65 **	62 **	1:2.6	55	TSCC (71%), BTSCC (58%), other (17%)	HPV DNA and p16 (>75%)
ltaly, 2020 [32]	Italian Cancer Institute	2010–2019	339	-	-	61	1:3.5	48	TSCC (58%), BTSCC (49%), other (20%)	HPV DNA and p16 (>75%)
Sweden, 2019 [33]	Stockholm	2000–2016	795	-	-	-	1:3	70	TSCC (74%), BTSCC (63%)	HPV DNA and p16 (>70%)
Spain, 2020 [34]	Catalan Institute of Oncology-Bellvitge, Hospital, Hospital del Mar, Hospital Parc Taulí and Hospital de la Santa Creu i Sant Pau	1991–2016	864	59 *	-	60 *	1:8	10	TSCC (15%), BTSCC (8%), others (3%)	HPV DNA and p16 (>70%)
Netherlands, 2018 [35]	VU University Medical Center	2000–2015	926	59	61	-	1:2	30	TSCC (39%), BTSCC (38%), soft palate, uvula, other (8%)	HPV DNA and p16 (staining not specified)
Greece, 2017 [36]	St. Svvas Regionl Anticancer Oncology University of Athens	2013–2015	22	-	-	59	1:2	18	TSCC (27%), BTSCC (14%), soft palate (0%)	HPV DNA
Croatia, 2019 [37]	University hospital Center Zagreb	2002–2015	99	60	60	60	1:4	29	TSCC(43%), BTSCC (45%) soft palatae, other (17%)	HPV DNA and HPV RNA
Italy, 2020 [38]	Treviso Regional Hospital, Hospital of Mirano, and Trieste Cattinara Hospital	2000–2018	130	-	-	65	1:3	32	TSCC (26%), BTSCC (28%), other (5%)	HPV DNA and p16 (>70%)
Germany, 2019 [39]	ENT-Giessen	2000–2017	730	-	-	-	-	27	-	HPV DNA and p16 (>70%)
Kazakhstan, 2018 [40]	Kazakh Institute of Oncology and Radiology	2015–2017	35	-	-	-	1:2.5	26	-	HPV DNA and p16 (>70%)
Scotland, 2019 [41]	West of Scotland Cancer Network	2013–2015	229	-	-	60	1:3	60	-	HPV DNA

HPV: human papillomavirus, OPSCC: oropharyngeal squamous cell carcinoma. * mean age, ** data covering only study period 2015–2017.

## Data Availability

Not applicable.
